# CFD simulation and experimental validation of in‐container thermal processing in Fesenjan stew

**DOI:** 10.1002/fsn3.2083

**Published:** 2020-12-27

**Authors:** Maryam Soleymani Serami, Yousef Ramezan, Morteza Khashehchi

**Affiliations:** ^1^ Department of Food Science and Technology Faculty of Pharmacy Tehran Medical Sciences Islamic Azad University Tehran Iran; ^2^ Nutrition & Food Sciences Research Center Tehran Medical Sciences Islamic Azad University Tehran Iran; ^3^ Department of Agro‐Technology College of Aburaihan University of Tehran Tehran Iran

**Keywords:** CFD, conductive heat transfer, Fesenjan stew, in‐container sterilization, optimization, retort

## Abstract

The purpose of the study was to develop and validate the CFD model for predicting temperature profile and determine the slowest heating zone (SHZ) during the thermal processing of Fesenjan stew. The temperature profile was recorded experimentally at a point where it was predicted to be the SHZ. The results showed that the SHZ was located at the geometric center of the containers, and the temperature reached 120.3 °C. The average temperatures of experiment versus predicted temperatures at the same positions, no significant difference were observed (*p* < .01). The RMSE calculation showed good agreement between simulation and experimental data (RMSE = 1.36). The container geometrical center F_0_ was measured 17.68 min and indicated that an additional process was applied. An 11.5 min reduction in holding time was estimated to optimize the process since the F_0_ of some meat and sauce‐based food is about 6–7.65 min.

## Graphical abstract

SHZ was located in the geometrical center of the container. CFD can predict accurately the temperature profile in the SHZ of Fesenjan stew. The experimental results showed that overprocessing is applied to the product.

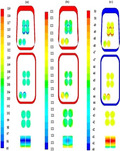

## 1 INTRODUCTION

In the food industry, the steps of the process that leads to eliminating foodborne disease are called conservation (Singh & Heldman, 2009). The thermal process deactivates bacterial spores in cans, using temperatures above the boiling point of water by high‐pressure water vapor retorts (Farazbakht et al., 2017). The most critical concern in the canned food products industry is the destruction of *Clostridium botulinum* bacteria, especially in low‐acid foods (pH > 4.5). Spores of this bacterium are resistant to heat, and if allowed to grow, produce a deadly toxin (Padmavati & Anandharamakrishnan, 2013).

Commercial sterilization is an intense heat process to reduce the population of all types of microorganisms in a product (Ghani et al., 1999; Vatankhah et al., 2015). Usually, the temperature used for the sterilization process is higher than the boiling point of water. This process is generally carried out by the steam, to heat the food inside the can to a specified temperature, then holding at that temperature for a sufficient time until the destruction of microorganisms (Varma & Kannan, 2006; Vatankhah et al., 2015).

The physical properties of the product and heating method determine the amount of heat required for the process. In the canned food industry, accurate analysis of the heat transfer mechanism will help improve food quality, optimize process conditions, and prevent energy loss (Vatankhah et al., 2015). Heat transfer mechanisms in food containers are conduction for solids and high viscosity liquid foods, natural convection for low viscosity liquid foods, convection, and conduction for liquid foods with solid particles and convection and then conduction for liquid foods which contains starch or viscosity improvers (Cordioli et al., 2015; Dimou et al., 2011). In foods such as canned tuna, thick syrups, purees, and concentrates, conduction is considered as the only way of heat transfer (Cordioli et al., 2015; Kızıltaş et al., 2010).

Thermal processing is one of the essential methods of preservation of food with long shelf time. Proper use of heat can secure food microbiologically, but the quality of food, including its nutritional characteristics, is significantly reduced by keeping it at high temperatures (Cordioli et al., 2015; Dimou et al., 2014). Therefore, the combination of temperature and time during the heating and cooling period of the product should be carefully evaluated to ensure both efficacy (inactivation of microorganisms and enzymes) and efficiency (protection of sensitive and limiting nutritional factors) (Farazbakht et al., 2017). Designing thermal processes is done by carrying out numerous experiments with high time and cost, thus reducing the possibility of achieving fast, practical, and profound results (Cordioli et al., 2015; Ghani et al., 1999).

To overcome these restrictions, in recent years, process design in the food industry has increasingly been done by numerical solutions of process governing equations, modeling, and computation. Among these methods, CFD has been widely used in many parts of the food processing (Cordioli et al., 2016).

CFD is a simulation tool that uses powerful computers and mathematics to model fluid flow conditions to predict heat, mass, momentum, and optimal design of industrial processes (Cordioli et al., 2015; Park & Yoon, 2018).

CFD recently has been used in the food industry. The success criterion has matched the rate of simulation results with experimental results. This method has attracted much attention from the international community as a developing knowledge (Cordioli et al., 2015).

The SHZ is a region within a container that takes the most time to reach the final temperature of sterilization and therefore limits the speed of processing (Padmavati & Anandharamakrishnan, 2013; Park & Yoon, 2018; Varma & Kannan, 2006). The SHZ and slowest cooling zone (SCZ) have no fixed location inside the container. In most food products, these points change with the heating transfer mechanism involved in the process. It is possible to precisely monitor the movements of SHZ and SCZ during the whole thermal processing using the developed CFD models and considering the points with the lowest and highest temperature in the heating and cooling phase, respectively(Cordioli et al., 2016; Rinaldi et al., 2018).

The purpose of this study is to determine the temperature distribution in the SHZ and review the retort settings in order to prevent loss of thermal energy, time, and nutritional values. Also, a CFD model was developed and validated to predict the temperature profile during the retort heating process, in commercial Fesenjan stew packaged in the semirigid aluminum containers.

## 2 MATERIALS AND METHODS

### 2.1 Material

The Fesenjan stew was prepared and packaged in a semirigid aluminum container by the Hani Foods Company for this project. The size of a rectangular cube shape container was 135 × 25 × 100 mm. All of the chemicals used in this study have been purchased from Merck (Germany).

### 2.2 Methods of experiments

Moisture, protein, fat, and ash were measured by AOAC official method (Helrich, 1990). The total carbohydrate content was calculated based on the method mentioned in CAC/VOL IX‐Ed.1, Part III (CAC, 2015). Fiber measurements were carried out by the ISO method (Determination of crude fiber content. General method ICS: 67.050 General methods of tests & analysis for food products, 1981). The pH was measured after the preparation of the Fesenjan stew using a standard digital pH meter (HI98161 Hanna Company). The pH meter was calibrated before the test using buffers 4 and 7. The pH was measured after sample homogenization. Density measurements were performed using a pycnometer at 80 °C.

### 2.3 Governing equations

#### 2.3.1 CFD main equation

The fluid condition is related to a group of cells, where all the active equations are solved, and it is necessary to determine their physical properties. The solid region is a bunch of cells that the heat conduction equation is the only equation that is solved for them. No equation for fluid flow was used in the solid state. Materials may behave like solid, although they are fluid. For these materials, it is assumed that there will be no convection. To solve the energy equation in geometry and the boundary conditions associated with that ANSYS Fluent 16 software were used. The energy equation was solved using Equation 1.

(1) $$\left(\frac{\partial \rho h_{\text{total}}}{\partial t}‐\frac{\mathrm{Dp}}{\mathrm{Dt}}+\nabla \cdot \left(\rho Vh_{\text{total}}\right)\right)=\nabla \cdot \left(k\nabla T\right)$$

In this equation, *h*_total_ is special total enthalpy based on temperature and pressure (Pa), *ρ* is density (kg/m^3^), t is time (s), p is pressure (Pa), V is volume (m^3^), K is thermal conductivity (W/m °C), and T is temperature (°C), since there is no internal energy source.

### 2.4 Calculation of thermo‐physical properties of each component

Physical properties such as specific heat, and thermal conductivity, density are usually assumed to be constant due to small variations about temperature. In this study, the mentioned properties were measured at room temperature (25 °C) according to the Ghani and Farid method (2007) (Ghani & Farid, 2007).

The thermal conductivity and specific heat were estimated by the mass fraction of their constituents (water, fat, protein, ash, and carbohydrate) (Sahin & Sumnu, 2006).

The thermal conductivity was calculated based on Equation 2:

(2) $$k=\sum_{i=1}^{n}k_{i}Y_{i}$$

where a composition is composed of n different compounds, K_i_ is the thermal conductivity of the i_th_ compound (W/m °C), and Yi is the volumetric component of i_th_ that is obtained using Equation 3:

(3) $$y_{i}=\frac{X_{i}/\rho_{i}}{\sum_{i=1}^{n}\left(X_{i}/\rho_{i}\right)}$$

where *X*_i_ is the mass component, and *ρ*_i_ is the density of the i_th_ compound.

The specific heat was calculated using the Equation (4):

(4) $$C_{p}=\sum_{i=1}^{n}C_{\mathrm{pi}}X_{i}$$

where C_pi_ (kJ/kg °C) is the specific heat, and X_i_ is the mass component of ith compound.

### 2.5 Model validation and error evaluation

The root mean square error for the comparison of measured and simulated temperatures was calculated using Equation 5.

(5) $$\text{RMSE}=\sqrt{\frac{1}{n}\sum_{i=1}^{n}{\left(T_{\exp}‐T_{\text{sim}}\right)}^{2}}$$

where RMSE is the root mean square error, T_exp_ is the temperature measured experimentally (°C), and T_sim_ is the simulation temperature (°C).

### 2.6 Calculation of thermal death index or sterilization value (F_0_)

The sterilization value is obtained from Equation 6:

(6) $$F_{0}=\int_{0}^{t}10^{\left(T‐121.1\right)/z}dt$$

F_0_ is the sterilization value at 121.1 °C, T is the predicted temperature or temperature measured in the SHZ (°C), and Z is the thermal resistance coefficient, which is equal to 10 °C (Singh & Heldman, 2009).

### 2.7 Fesenjan stew preparation method

**FIGURE 1:**
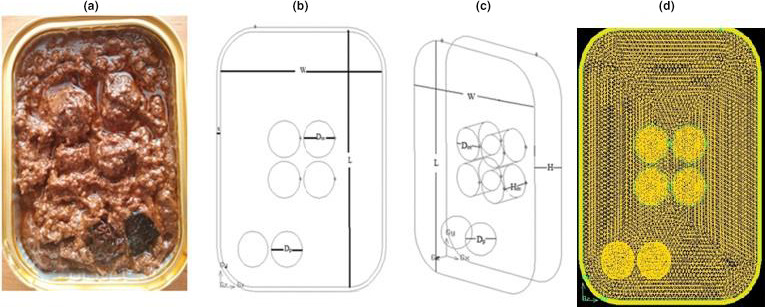
The structure and dimensions of the container and the location of the components in the container. (a) Horizontal view of the filled container, (b) the shape of the horizontal view of the container, W, width, L, length of the container, D_m_, the diameter of the meatball, and D_p_ of the diameter of the Bukhara Plum, (c) three‐dimensional schematic drawing of the container, W, width, L, length, and H, height of the container, D_m,_ diameter of the meatball, H_m,_ height of the meatball, and D_p,_ diameter of the Bukhara plum, (d) horizontal view of geometry and its meshing

Fesenjan stew is a traditional Iranian stew. This stew has consisted of stew sauce, pieces of ground meat, was named "meatballs" and Bukhara plums with weight percentages of 67, 22, and 11%, respectively. A semirigid aluminum container that is commonly used for packaging such foods was used in the form of a rectangular cube. Figure 1 shows the structure and dimensions of this container and the location of each component of the stew inside the container.

In each one of containers, four pieces of meatballs with a total weight of 40 g in the center of the dish and two Bukhara plums with a total weight of 20 g were placed on the side of the container. Meatballs were a combination with a specific composition of beef and sheep ground meat, onions, and spices. The mixture was formed into a cylindrical geometry. These meatballs were boiled in water for 5 min. Their temperature reached 40 °C when the containers were filled.

Then, the dishes were filled with Fesenjan sauce. The sauce included walnut, apple, onion (all of which were ground), concentrates of pomegranate, tomato paste, oil, water, other spices, and seasonings. The sauce was boiled for 2.5 hr and reached the desired concentration. After filling, containers were sealed and loaded into baskets and transferred to the retort.

### 2.8 Experimental study of temperature

The thermal processing of Fesenjan, considering commercial sterilization conditions, was investigated in a semirigid aluminum container. The steam and hot water are sprayed from the nozzles located on the top of the baskets containing stews inside the retort (Barriquand, France). The retort temperature was adjusted to 121.1 °C. The samples were placed on the middle floor of two baskets located in the middle of the retort. As usual, the capacity of baskets and retort was filled with similar containers and contents.

Three filled containers were used to obtain an average temperature, and then, the containers were sealed at 280°C using an Alcan machine. In each container, type *K* thermocouple (The probe contained a hypodermic needle with 36 mm long and 0.5 mm diameter, having a 0.05 mm diameter constantan and a thermocouple) was placed at 67.5 mm (sauce between pieces) to measure the temperature during the thermal process in the center of the container where was expected to be the SHZ. Another thermocouple was devised near the containers, in the water cascading Barriquand steriflow (Roanne, France) retort.

The thermocouple was connected by a cable (PT 100) to a 4‐channels data logger (Ellab CTF 9004 – France). In order to record temperature, the data logger was connected to a personal computer on which the E Val 2.1 software was installed. The measured temperatures in each of the containers were recorded at 60 s intervals. The average temperature at the SHZ of the three containers reached a temperature of 120.3 °C. The temperature was recorded until the end of the cooling step and reaching the temperature of 80 °C (Appendix S1).

### 2.9 Optimization

One of the issues that are very necessary to be considered is the problem of overprocessing of canned products in the food industry. In many studies on the adequacy of the thermal processing of canned food in Iran (Farazbakht et al., 2017; Shafiekhani et al., 2016; Vatankhah et al., 2015), this problem is seen, which has been happened in this study too. As mentioned before, in order to ensure the correctness and adequacy of the thermal process, the meatballs were placed in the center of the container. Therefore, although F_0_ is not defined explicitly for Iranian foods, it can be compared with the F_0_ of meat, which is defined about 6–7.65 min (Noronha et al., 1996).

### 2.10 Simulation of process

#### Geometry details and meshing

Geometry making and meshing have been done using GAMBIT2.4.6 software. The container's geometric shape was constructed according to its actual shape, like a rectangular cube of 135 × 25 × 100 mm, and then at its angles along the z‐axis, arcs were created with a radius of 21.5 mm. The 8 pages that formed the wall of the container became like a single page. The geometric shape of meatballs was cylindrical with dimensions of height and diameter of 19.4 and 18.6 mm. The geometric shape of the plum was made as a sphere with a radius of 4.9 mm. Meatballs and plums were moved to the place wherein the actual conditions they were located (meat in the middle and plums on the container's side). Meatballs and plums were spaced as much as 1.5 mm from the bottom of the container in order to be tangent to the bottom of the container and allow proper meshing. For stew sauce volume creation, the volume of meatballs and plums was subtracted from the container's total volume (retaining the volume of components and not retaining the original volume). At the first meshing was performed for each page and then for volumes (Figure 1d). In order to verify more precisely the slowest heating zone, a finer mesh was chosen for meatballs, which were placed at the geometric center of the container. Finally, 111,441 nodes were used, and total treatment time discretization was precisely the same in the experimental processing (Come up time: 25 min, holding: 42 min, and cooling: 42 min).

#### Boundary conditions

Generally, simulation is based on uniform heat and constant temperature in all directions of the container (Varma & Kannan, 2006). In defining the boundary conditions ceiling, walls, and floor of the container, the surface of meatballs and plums are defined as walls. For walls, ceiling, and floor of the container, the characteristics of momentum and slip are defined as stationary walls and nonslip. The thermal conditions of the walls of Bukhara plums and meatballs, which were placed between the two materials, were defined as a couple. The temperature of each component inside the container is different at the starting point, and with the patch option, the initial temperature is determined for each one.

$$\text{At t}=0\;T_{w}=37^{\circ }C,\;T_{s}=55^{\circ }C,\;T_{p}=25^{\circ }C\;\&\;T_{m}=40^{\circ }C$$

It is assumed that the thermal media supply temperature of 121.1 °C all over the container, and due to the insignificant thermal resistance of the container, the boundaries of the stew sauce will also reach the same temperature without delay.

The method of calculation and Solution methodology was implicit. In the formulation of solving the conditions of pressure based, implicit, transient, second‐order implicit equations and laminar physical models were used. Energy equation activated. In material defining, the properties of stew sauce, meatball, and Bukhara plum according to the characteristics listed in Table 1 were defined in a new database. The aluminum packaging properties were selected from the list of materials in the program database. The starting point of the solution was from the walls of the can. At the first 25 min, when the retort temperature was rising, at the end of every 60 s, the temperature of the container walls changed, assuming that the aluminum wall temperature reached the retort temperature at the same time, and the rest of the conditions remained unchanged. At the constant retort temperature, the wall temperature of the container was 121.1 ºC. This solving condition continued for 42 periods of 60 s. After the cooling phase started, 42 periods of 60 s were considered. At the end of each 60 s, the temperature of the walls of the container was changed, and at the end of the 109 min and the temperature reached 40 °C, similar to the production conditions, the problem solving has been finished.

**TABLE 1:** Thermo‐physical properties of different mass components and various components of stew at 25 °C

	*ρ* (kg/m^3^)	k (W/m °C)	C_p_ (J/kg °C)
Protein	1,288.43	0.26	2,097
Fat	892.18	0.16	2,071
Carbohydrate	1,574.26	0.28	1,668
Fiber	1,282.23	0.26	1,963
Ash	2,401.35	0.42	1,220
Water	973.38	0.67	4,204
Meatball	1,046	3,230	0.4
Plum	1,378	2,525	0.45
Stew sauce	1,058	2,800	0.35

## 3 RESULTS AND DISCUSSION

**FIGURE 2:**
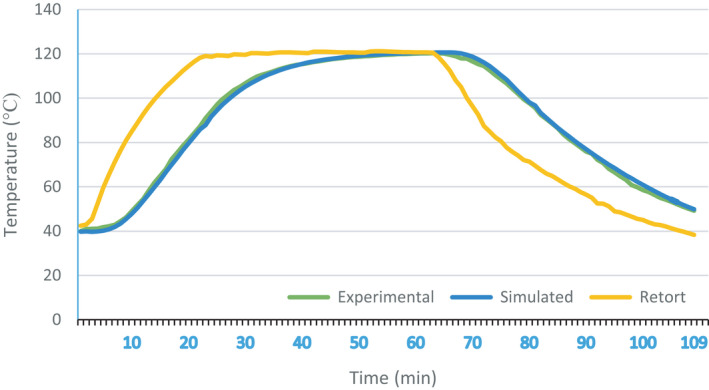
Time–temperature profile of retort. Results from experimental measurements and simulation results

The thermo‐physical characteristics of each component are calculated by considering the specific formula for each of them. The results of these calculations are presented in Table 1. These results were used as inputs in the definition of ANSYS Fluent software. The ability of this software to predict the location of the SHZ has been proven in other studies that have been done for this purpose (Cordioli et al., 2015; Dimou et al., 2011; Ghani et al., 1999, 2001; Padmavati & Anandharamakrishnan, 2013), and in this regard, the results of this study are consistent with the studies mentioned. From the comparison of the measured values of the experimental method with the model temperature profile at the location of the thermocouple during the process, the validity of the proposed model was confirmed. Figure 2 shows the temperature chart relative to the retort time for the results of the experimental measurements and the results of the simulation. As shown in the diagram, in the first three min of the process, the three temperatures are close together, and with little difference, which increases with the progress of the process, this is due to the initial temperature of meatballs and stew sauce. In fact, in the first 6 min, the retort temperature is lower than the temperature of sauce; during this time, the initial temperature of the sauce increases the temperature of meatballs, Bukhara plums, and the walls of the container. As it is distinguishable from the chart, during this period, the temperature rise rate is prolonged. After the retort temperature rises, all the components of the container, the retort, and the SHZ temperature will continue to be about the same. At the end of the 25th min, the retort enters the holding phase, while due to the difference between the wall of the container and its other parts temperature, the heat transfer in the container continues. However, gradually and by reducing the difference, the line slope decreases.

At the end of the 65th min, the retort enters the cooling phase. However, at the same time, the temperature increase in the center of the container continues slowly until the 70th min. After this time, the temperature in the center of the container was reduced, although the slope decreases slower than the retort temperature drop.

**FIGURE 3:**
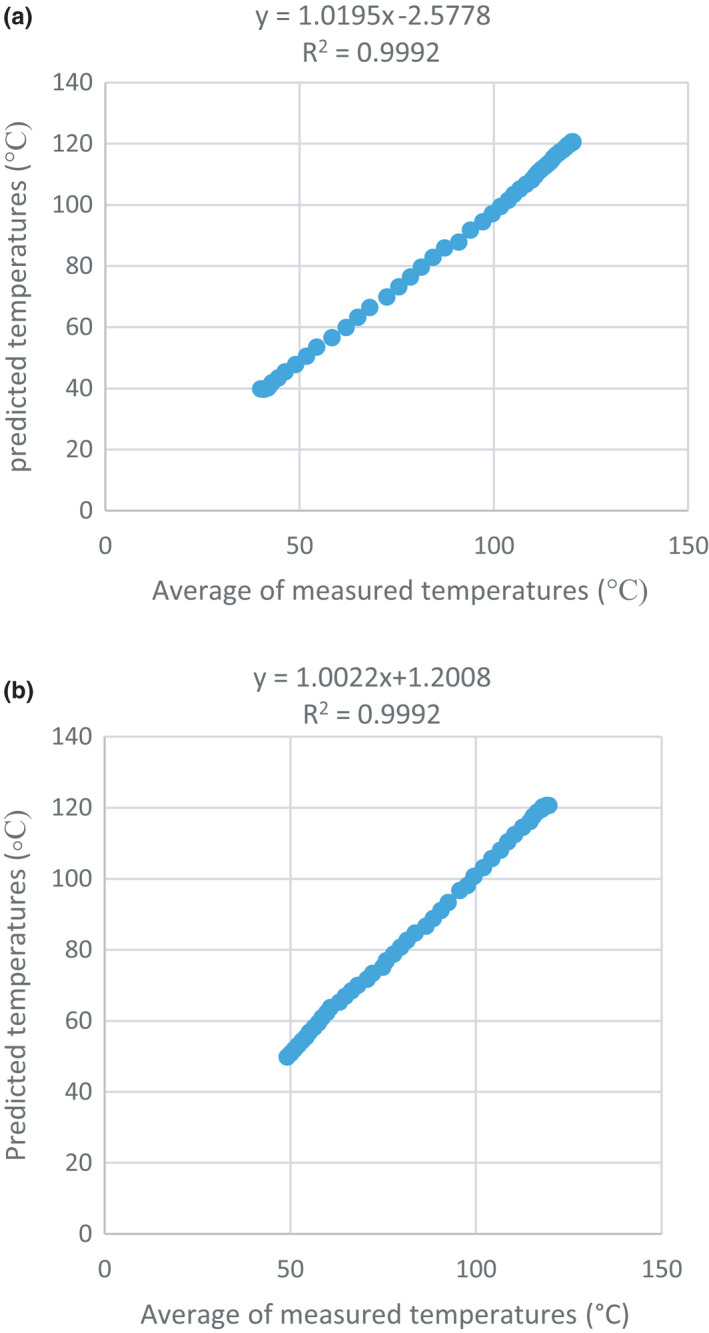
Comparison of the predicted temperature by simulation and measured temperature (a) during the heating period and (b) during the cooling period

The data from the simulation and experimental measurements of temperature were divided into two groups, including the temperature rise period (from the beginning of the process to the end of the holding time) and the temperature reduction period (cooling process). Figure 3 shows the result of this comparison as two graphs. In the best case, that is, the conditions in which the predicted temperature and the measured temperature are precisely equal to each other, these two graphs should have the same slope, and both create an angle of 45 degrees with the longitudinal axis. It is also seen that the results provided this object.

**FIGURE 4:**
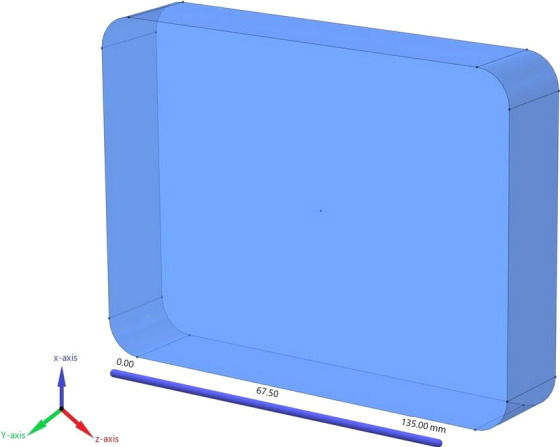
Three‐dimensional schematic drawing of the container

**FIGURE 5:**
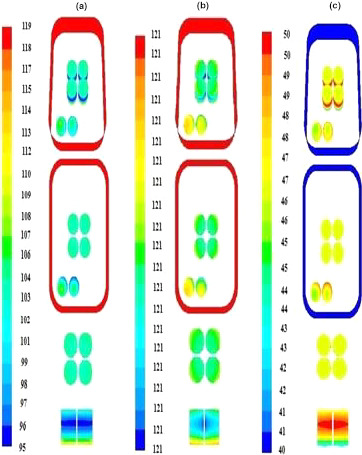
Temperature Contours (a) at 25 min (start of holding time), (b) at 65 min (end of holding time), and (c) at 109 min (end of cooling time)

The study of temperature at different time intervals and points of the container indicates that the SHZ was located at the geometric center of the container (*x* = 50, *y* = 14.2, *z* = 67.5 mm) (Figure 4). As mentioned in the materials and methods section, meatball pieces were located in this part of the container, and the SHZ was located in the upper half of them (at the height of 57% from the bottom of the container). In Figure 5, the temperature contours are shown in several different time steps.

Earlier researches conducted on sterilization of solid foods such as canned tuna, purees, thick syrups, and concentrates were assumed to be through conduction heat transfer only, and similar to this study, SHZ was located at the geometric center of the container (Cordioli et al., 2016; Rinaldi et al., 2018; Varma & Kannan, 2006). In studies conducted by Vatankhah et al. (2015), Vatankhah et al. (2016), they found out that SHZ locations in Haleem and apple puree were packaged in semirigid aluminum containers, remained fixed at the geometric center of the container(Vatankhah et al., 2015, 2016).

The results of this research are in complete agreement with our previous study. Celery stew was packaged in a semirigid aluminum container. Experimental data and simulation models showed that SHZ was located at the geometrical center of the container. Thermal processing time was calculated according to SHZ due to prevent overprocessing and optimize the process (Berenjforoush Azar et al., 2020).

### 3.1 Calculation of root mean square error

The RMSE was calculated by Equation 5, to compare the average of the measured temperature and the temperature calculated by the software; this value is equal to 1.36. This small amount of error indicates the validity of the simulation method by the experimental method, and it can be concluded that the two methods are highly consistent with each other.

### 3.2 Sterilization value (F_0_)

The sterilization value or thermal death index was calculated for two sets of information, including simulated temperatures and measured temperatures in the experimental method. The equation was solved by inserting the temperature at the end of each min of holding time instead of T and then calculating the total time (Equation 6). Student's *t* test was used to compare simulated and experimental data. The results showed a high similarity between the predicted and experimental values at the probe position point for both temperature rise and reduction (α = 0.01). The F_0_ calculated by the experimental and simulation methods was equal to 17.68 min (Figures 2 and 3).

### 3.3 Process optimization

The coldest zone at the end of the heating period was chosen to be studied for sterilization efficiency. Results showed 17.68 min as total F_0_. The retort temperature program was adjusted to process some Iranian Stews. According to the stew formula, Fesenjan contains meatballs, Bukhara plums, and sauce (pH = 4.6). The F_0_ was compared with meat‐based formulations because meatballs were the biggest particle in the Fesenjan stew and placed in the container's geometrical center (SHZ zone). The amount of F_0_ for the meat‐based formula is 6–7.65 min (Berenjforoush Azar et al., 2020; Noronha et al., 1996; Sun, 2012).

**FIGURE 6:**
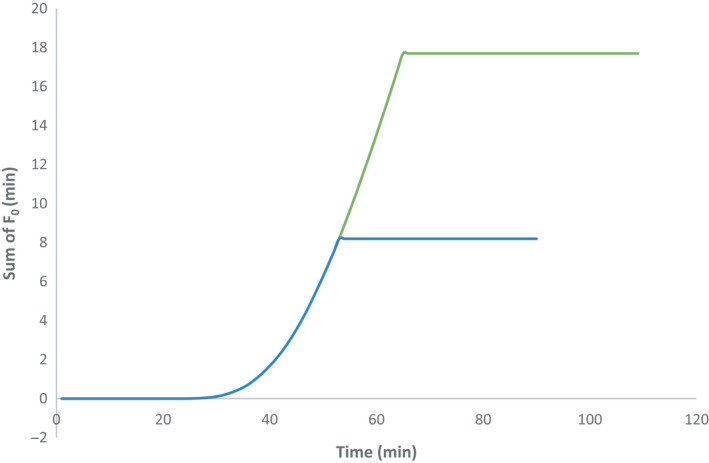
Comparison between the process F_0_ and the new settings of retort, the green line (−) represents the sum of the index F for the initial settings of the retort and the blue line (−) Total F index for the new settings of retort

Thus, the time–temperature was adjusted to reach a lower value of F_0_ by using Simpson's rule for the numerical approximation of integration (Alonso et al., 2013; Holdworth, 1993). Results showed that the product would reach logical sterility about F_0_ = 6.1 min., by decreasing 11.5 min of the heating period (Figure 6). Therefore, in order to maintain the quality and nutritional value and also in order to save energy, it is essential to review the retort settings and optimize the process.

## 4 CONCLUSION

A mathematical model was tabled to study the temperature distribution and sterilization of Fesenjan in semirigid containers and validated using experimental methods. As shown by the experimental and simulation results, the calculated F_0_ from the two methods were covered each other in the whole processing time. Also, the RMSE represents a small amount of error. These results indicate that the two methods are highly consistent with each other. This adaptation is promising to use a new research methodology in the industry and research, with which accurate results can be obtained without spending too much time and cost of experimental work.

The relationship between the temperatures obtained from the simulation and the experimental temperatures shows a linear relationship, and the proximity of the coefficients of determination for heating and cooling stages were equal to 1 (R^2^ = 0.999), indicates the correctness of the simulation.

The main objects set for this study were the adaptation of the CFD method with the experimental method, validation of the CFD method, and the review of sterilization adequacy. From the comparison of measured values with the model temperature profile at the location of the thermocouple during the process, the validity of the proposed model was confirmed. In the many studies that have been done on the thermal processes of food products in Iran, the overprocessing is a significant problem, because of the cheap energy costs in Iran and the concern of manufacturers for nonsterilizing products. While today not only the world's energy price is much higher than it is in Iran, but also energy is a critical topic, and much attention has been paid to the proper use of these finite sources. In order to save energy, improve the nutritional values and organoleptic properties of this stew, it is suggested that the retort time–temperature settings be readjusted.

## Nomenclature

CFD: Computational fluids dynamics
Cp: Special heat (kJ/kg °C)
F_0_: Sterilization value (s)
h_total_: Special total enthalpy (J/kg)
λ, k: Thermal conductivity (W/m °C)
RMSE: Root mean square error
S_E_: Internal energy source (J)
T: Temperature (°K)
t: Time (s)
T_exp_: Experimental temperature (°C)
T_sim_: Simulated temperature (°C)
Tw: Wall temperature (°C)
X: Mass component
Y: Volumetric component
Z: Thermal resistance coefficient (°C)
ρ: Density (kg/m^3^)

## Supporting information

App S1
