# Supplementary figures and images for: CFD simulation and experimental validation of in‐container thermal processing in Fesenjan stew
Source: Food Sci Nutr. 2020 Dec 27;9(2):1079–87. doi: 10.1002/fsn3.2083 (PMC7866580; doi:10.1002/fsn3.2083)

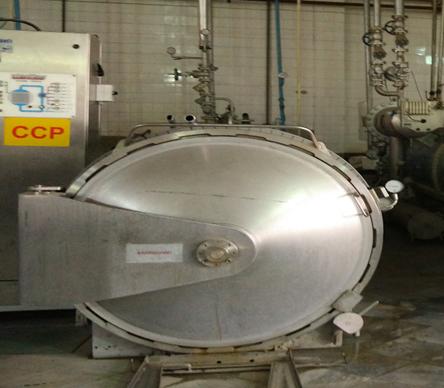


Horizontal static retort


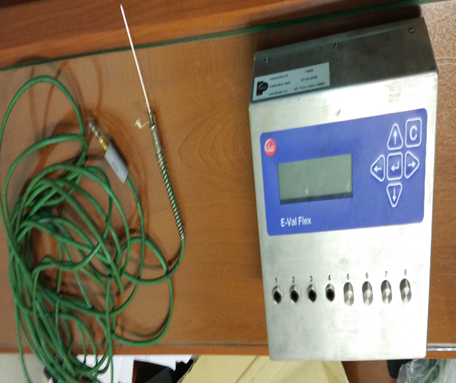


Data logger and type K thermocouple


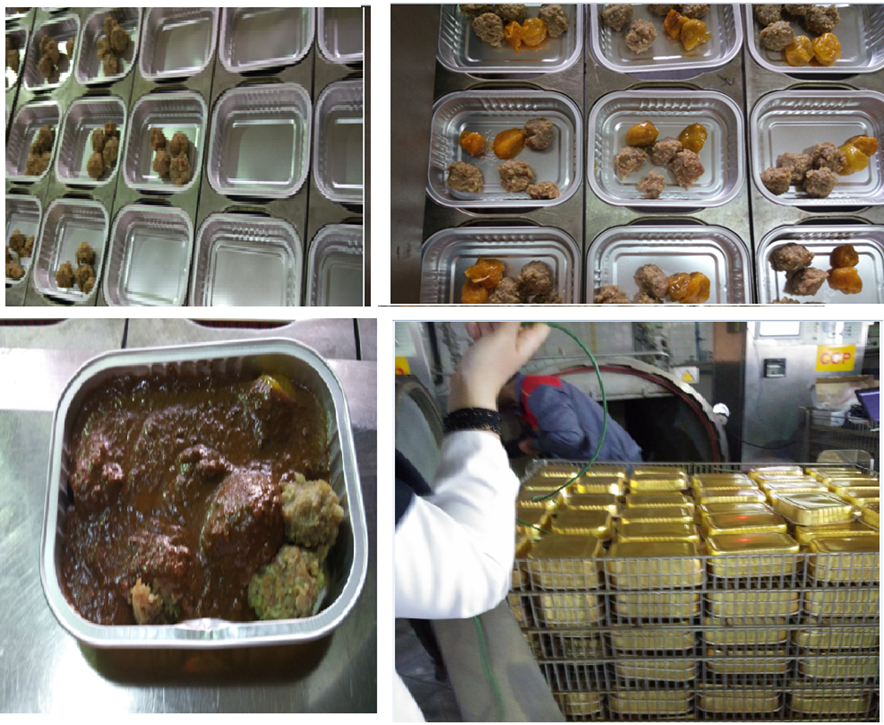


Fesenjan product line

Supplement: Supplementary file 1 — App S1 [file FSN3-9-1079-s001.doc]
